# Supplementary material for: Prevalence and factors associated with puerperal sepsis among postnatal women at a Tertiary Referral Hospital in Western Uganda
Source: PLoS One. 2025 Aug 4;20(8):e0311708. doi: 10.1371/journal.pone.0311708 (PMC12321055; doi:10.1371/journal.pone.0311708)
Supplement: Attachment — Submitted filename: Response to reviewers.docx [file pone.0311708.s002.docx]

Moses Asiimwe

Mountains of the Moon University

Uganda

[mosesae19@gmail.com](mailto:mosesae19@gmail.com)

June 18, 2025

The Editor,

Plos One

Dear Editor,

**Re: Submission of our revised manuscript titled; Prevalence and Factors Associated with Puerperal Sepsis Among Postnatal Women at Tertiary Referral Hospital in Western Uganda**

I hereby submit to you our revised manuscript for your review and publication in Plos one journal. Our study aimed at determining the Prevalence and Factors Associated with Puerperal Sepsis Among Postnatal Women at Tertiary Referral Hospital in Western Uganda.

Our study aimed at generating this evidence to promote the implementation of evidence-based policies and programs in our settings and the country.

This paper communicates to a wider scientific and public audiences, policy makers and researchers about the opportunities and challenges in promoting Maternal health to reduce mortality and morbidity due to post-partum infections.

In our revised submission, we add a statement to our financial disclosure that; **The funder had no role in study design, data collection and analysis, decision to publish, or preparation of the manuscript**.

Thank you

Moses Asiimwe

Lecturer, Public Health

Mountains of the Moon University

**Matrix of comments**

Title: Prevalence and Factors Associated with Puerperal Sepsis among Postnatal Women at a Tertiary Referral Hospital in Western Uganda

| **SN** | **Comment** | **Response** | **Page number/section** |
| --- | --- | --- | --- |
|  | Lines 116-117: All the postnatal hospital records for the previous six months prior data collection were identified and we selected all files that fulfilled the inclusion and exclusion criteria  Comment: The methods section in abstract mentioned that patient files of 180 postnatal mothers who were admitted at Fort Portal Regional Referral Hospital from 20 February, 2024 to 01 April, 2024 were included in the study? Please explain inclusion and exclusion criteria in study population section  . | Thank you so much for this comment, the inclusion and the exclusion criteria has been included in the population section | Please see lines 111 to 112 |
|  | 129-131: Puerperal sepsis was considered in this study as a mother with a laboratory-based diagnosis with positive bacterial culture results during the course of postnatal admission  Comment: Any abnormal vital signs threshold, SIRS, qSOFA or SSC criteria were considered for diagnosis? | Thank you so much for this concern, however in our study we only used the positive bacterial culture results to define puerperal sepsis |  |
|  | Line: 134 Data collection was conducted between 20 February 2024 and 01 April, 2024.  Comment: Is this the data collection period or patient admission period? Please refer to methods section  Clinically | Thank you for this response, however the period of 20^th^ of February 2024 to 01^st^ of April 2024 was the data collection period. The admission was all those mothers who admitted in postnatal 6 months prior to data collection as indicated in the study population section | 108 to 112 |
|  | 151-152: plausible variables and those with a p value of ≤0.2 at bivariate analysis were taken for multivariate analyses to adjust for confounding.  Comment: plausible variables and those with a p value of ≤0.2 at bivariate analysis were taken for multivariate analyses to adjust for confounding variables | Thank you so much, this statement has been adjusted as per the guidance in the manuscript | Line 153 |
